# Is Isotretinoin in Acne Patients a Psychological Boon or a Bane: A Systematic Review

**DOI:** 10.7759/cureus.16834

**Published:** 2021-08-02

**Authors:** Savitri Chandrasekaran, Joaquim Francisco Maria De Sousa, Smit Paghdar, Taheseen M Khan, Nishant P Patel, Nicholas Tsouklidis

**Affiliations:** 1 Medicine, Indira Gandhi Medical College and Research Institute, Pondicherry, IND; 2 Internal Medicine, California Institute of Behavioral Neurosciences & Psychology, Fairfield, USA; 3 Medicine, S.S Institute of Medical Sciences and Research Centre, Davangere, IND; 4 Emergency Medicine, Healthway Hospitals, Goa, IND; 5 Internal Medicine, Surat Municipal Institute of Medical Education and Research (SMIMER), Surat, IND; 6 Medicine, Mahatma Gandhi Mission (MGM) Medical College, Navi Mumbai, IND; 7 Psychiatry, California Institute of Behavioral Neurosciences & Psychology, Fairfield, USA; 8 Internal Medicine, Government Medical College, Surat, IND; 9 Pediatrics, Wyckoff Heights Medical Center, Brooklyn, USA; 10 Health Care Administration, University of Cincinnati Health, Cincinnati, USA; 11 Medicine, California Institute of Behavioral Neurosciences & Psychology, Fairfield, USA; 12 Medicine, Atlantic University School of Medicine, Gros Islet, LCA

**Keywords:** acne, acne vulgaris, psychiatric effects, depression, anxiety, suicide, vitamin a, isotretinoin

## Abstract

Acne vulgaris is a frequently encountered dermatological condition in clinical practice. Isotretinoin is one of the drugs prescribed for this condition. However, it is unclear whether the use of this drug worsens or improves the psychological effects in an acne patient and whether it is advisable to use this drug commonly. This systematic review aims to assess the relationship between Isotretinoin and psychiatric side effects in acne patients.

A literature search was conducted using PubMed, Cochrane, and Google Scholar databases in accordance with Preferred Reporting Items for Systematic Review and Meta-Analyses (PRISMA) guidelines. Articles published within the last 10 years were taken into account and a review was conducted on the relevant articles after critical appraisal.

Nine studies were finalized for discussion and out of the nine studies, two studies concluded that Isotretinoin could cause psychiatric effects. Five studies showed no association between them. Two studies unexpectedly found that psychiatric symptoms improved because of Isotretinoin use. Lack of adequate sample size and absence of randomized controlled trials are the limitations of this study.

To conclude, Isotretinoin can be prescribed as a treatment option for severe acne despite some evidence of link with psychiatric effects. However, bearing the side effects in mind, a detailed evaluation before initiating the drug and a thorough monitoring while using the drug should be done as a standard practice in order to be on the safer side.

## Introduction and background

Acne vulgaris

**Figure 1:**
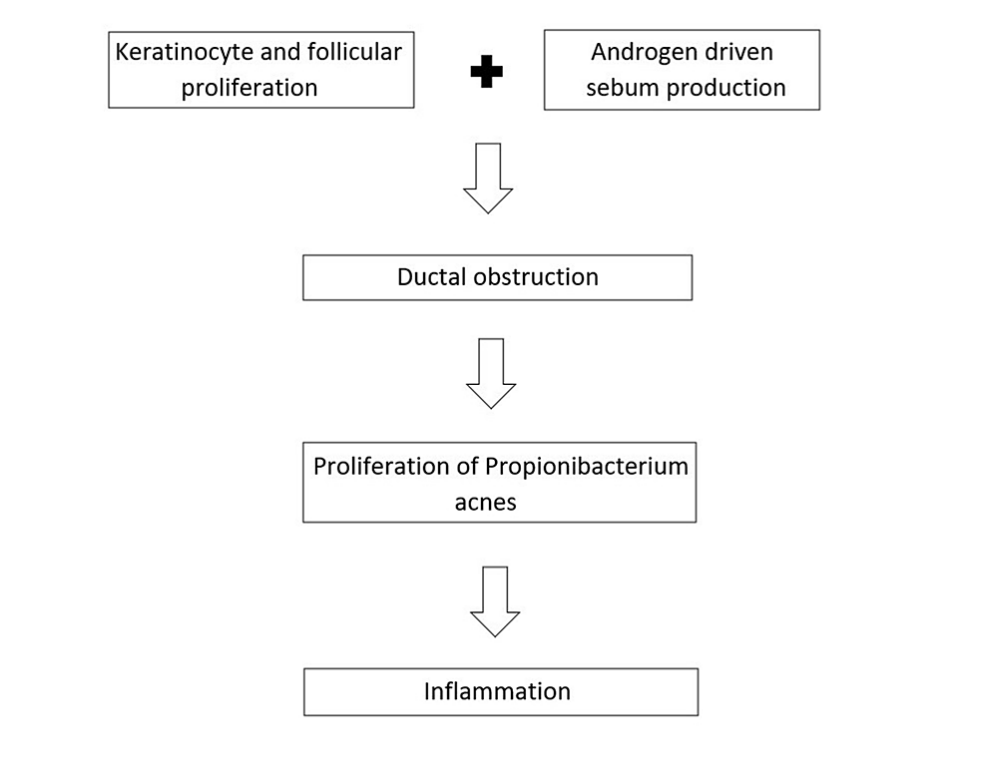
Pathogenesis of acne vulgaris.

Acne vulgaris is a common skin disorder caused by obstruction and inflammation of the pilosebaceous unit resulting from androgen-induced increased sebum production and bacterial colonization of hair follicles on the face, neck, chest, and back by a microbe called Propionibacterium acnes [1]. It is a prevalent chronic skin disease in the United States, affecting mainly adolescents and young adults [2]. It may generally present as comedones, papules, or pustules [3]. Besides leaving behind scars and affecting the quality of life, it may be associated with worrisome effects like depression, anxiety, and suicidal ideation [1,4,5]. The pathogenesis of acne vulgaris is depicted in Figure 1.

 

Isotretinoin

One of the most effective treatment modalities for acne is Isotretinoin, a first-generation retinoid approved by the Food and Drug Administration (FDA) in 1982 [6,7]. It is usually prescribed for severe nodulocystic or recalcitrant acne [2,8]. However, it has many adverse effects, out of which dryness of skin is reported to be the most common one [9]. Other documented side effects of this drug include teratogenicity, musculoskeletal effects (muscle stiffness, bone pain, and back pain), facial erythema, eye changes (dry eyes and blurry vision), effects on lipid levels, liver function tests, and psychiatric effects [6,9]. Due to the risk of teratogenicity, patients, pharmacists and prescribers must register with the U.S. Food and Drug Administration-mandated risk management program, iPledge, before starting isotretinoin therapy [2].

Few reported neuropsychiatric effects of Isotretinoin include depression, mood alterations, suicidal ideation, aggressive tendencies, anxiety, and psychosis [10]. Areas of the brain mainly prone to Isotretinoin are the hippocampus and prefrontal cortex [11]. They play a role in mood regulation and coordination of cognitive functions [11]. The property of Isotretinoin that enables it to cross the blood-brain barrier and affect such areas is its fat solubility [12].

The need for a systematic review

Similar to any other drug, Isotretinoin has its pros and cons. Hence, a dermatologist needs to assess whether the benefits of prescribing this medication outweigh the risks associated with it. Some studies propose that Isotretinoin might be associated with psychiatric side effects [6,7,9,10]. However, while further researching about this, it was found that the results from few papers show that there is no such significant association between the use of this drug and any neuropsychiatric effects [6,13-15]. The answer to whether the use of Isotretinoin in acne patients will cause harmful psychiatric effects or not remains unclear due to lack of complete and sufficient evidence.

Therefore, this research paper's primary purpose is to explore the association between the use of Isotretinoin and the risk of psychiatric effects among patients with acne by looking at different types of available studies. Furthermore, it attempts to clarify whether it is advisable to continue using this drug for acne, even if such adverse effects are proven to be there.

## Review

Methods

In this study, we followed and adapted the Preferred Reporting Items for Systematic Reviews and Meta-Analyses (PRISMA) guidelines. The main database used for literature search was PubMed, along with Cochrane and Google Scholar. The search was performed on January 23rd, 2021, using key phrases "acne OR acne vulgaris AND depression OR anxiety OR suicide AND vitamin A OR isotretinoin."

Various types of studies in which acne patients were given isotretinoin were included in our search. Using the MeSH keywords and phrases ("Depression"[Mesh]) OR "Anxiety"[Mesh]) OR "Suicide"[Mesh]) AND ("Acne Vulgaris/complications"[Mesh] OR "Acne Vulgaris/drug therapy"[Mesh] OR "Acne Vulgaris/psychology"[Mesh])) AND ("Isotretinoin/administration and dosage"[Mesh] OR "Isotretinoin/adverse effects"[Mesh])," twenty-eight relevant studies were found in PubMed.

Before screening and applying any inclusion/exclusion criteria, a total of 4,783 articles were found on PubMed. Duplicates were removed and after a review of titles and abstracts, 1,507 articles were chosen for further review. Once inclusion criteria were applied, studies found relevant included: (1) Full-text articles and (2) published within the last ten years. Then, a critical appraisal was performed using AMSTAR checklist and CARE guidelines. After applying inclusion criteria and performing quality check, studies amounted to nine.

**Figure 2:**
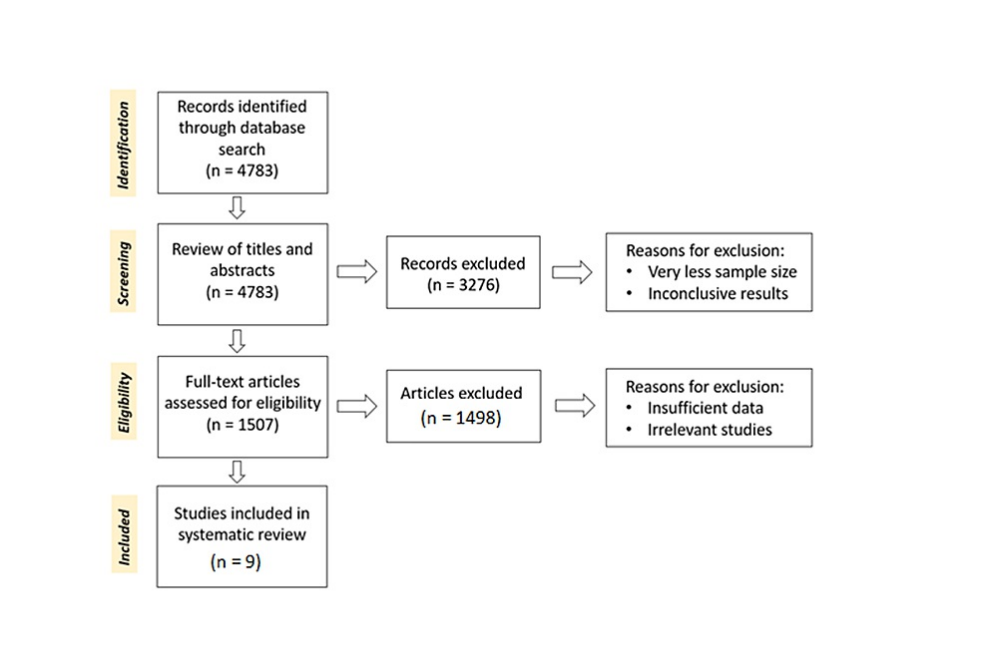
Study selection process.

Two authors independently assessed the articles for eligibility and extracted data from the selected articles. The following data were extracted from the included studies - author, location, study type, study period, study population, sample size, effects of isotretinoin on the patients, and assessment tools. Figure 2 shows the PRISMA flow diagram detailing study selection process.

Results

In total, nine studies explaining the relationship between Isotretinoin and psychiatric effects were included for discussion, which consisted of various types of studies.

The retrospective cohort study conducted by Sundström et al. in Sweden suggests a link between suicide and Isotretinoin [16]. The case-control study done by Droitcourt et al. in France also links isotretinoin usage to suicide attempts [17]. Although they are different kinds of studies, however, they propose that there must be some association between Isotretinoin and psychiatric features.

On the other hand, few studies suggest the contrary. Though conducted in different parts of the world, all these studies deny any association between Isotretinoin and psychiatric effects. For example, the prospective study by Erdoğan et al. concludes that Isotretinoin has no impact on depression, anxiety, or suicide [13]. The study conducted in Venezuela by Suarez et al. implies that Isotretinoin is safe to use with respect to psychological side effects [15]. The cohort and nested case-time-control study in France by Droitcourt et al. and the cohort prospective, questionnaire-based study conducted in Saudi Arabia by Algamdi et al. suggest that is no association between Isotretinoin and depression [18,19]. The systematic review and meta-analysis by Huang et al. in Taiwan also suggest the same [20].

Two studies even go ahead to suggest an improvement in psychiatric symptoms as a result of Isotretinoin usage. The systematic review and meta-analysis by Li et al. in China conclude that Isotretinoin improves depression symptoms in acne patients [21]. A prospective observational study conducted by Nikam et al. in India suggests that Isotretinoin causes an improvement in acne patients' anxiety [14].

The characteristics of the studies used for this review are included in Table 1.

**Table 1:** Description of the studies which met the inclusion criteria for this review.

Author	Location	Study type	Study period	Sample size	Conclusion
Sundström et al. [16]	Sweden	Retrospective cohort	1980-2001	57,56 patients	Increased risk of attempted suicide was apparent up to six months after the end of treatment with Isotretinoin
Droitcourt et al. [17]	France	Case-control	2010-2014	328,018 patients	Risk prone patients using Isotretinoin may attempt suicide
Erdoğan et al. [13]		Prospective, non-randomized, open-label study	Three months (2019)	102 patients	Isotretinoin did not affect the levels of depression, anxiety, and suicide in acne patients
Li et al. [21]	China	Systematic review and meta-analysis	Inception up to December 28th, 2017	20 patients	In acne patients, isotretinoin results in improvement of depression symptoms
Nikam et al. [14]	India	Prospective observational study	June 2017 to July 2018	300 patients	The use of Isotretinoin improved anxiety but had a negligible effect on depression
Suarez et al. [15]	Venezuela	A naturalistic, longitudinal, open-labeled study	February 2013 to June 2013	60 patients	Isotretinoin is safe with regards to psychological side effects
Droitcourt et al. [18]	France	Cohort and nested case-time-control study	2009-2016	443,814 patients	There was no association between Isotretinoin and suicide
Huang et al. [20]	Taiwan	Systematic review and meta-analysis	Inception to September 30th, 2016	31 patients	Isotretinoin use is not associated with depression
Algamdi et al. [19]	Saudi Arabia	Cohort prospective, questionnaire-based study	November 2019 to March 2020	29 patients	There is no direct relationship between the use of Isotretinoin and the development of depression

Discussion

Positive Association Between Isotretinoin Usage and Psychiatric Side Effects

A case-control study was performed by Droitcourt et al. in France from January 1, 2010, to December 31, 2014, with 328,018 subjects [17]. It studied the risk factors of suicide attempts in patients using Isotretinoin. The cases were patients with a suicide attempt during Isotretinoin use. The control group consisted of subjects exposed to Isotretinoin at the date of suicide attempt for the corresponding case. The cases and controls were matched for age, gender, and rank of the current isotretinoin course, and they were treated for the same duration. It was found that psychiatric history and history of anxiety alone were risk factors for suicide attempts in isotretinoin users. The study concluded that patients with risk factors at the time of treatment initiation did have incidents of a suicide attempt when using Isotretinoin.

The Swedish retrospective cohort study conducted by Sundström et al. had a sample size of 5,756, out of which 3,613 (63%) were males [16]. The study was conducted from 1980 to 2001. The age of the patients was between 15-49 years. All of them were prescribed Isotretinoin for severe acne. The mean length of treatment was 6.0 (SD 4.0) months for male patients and 6.1 (3.9) months for female patients. Two overall comparisons were made - (1) Study cohort was compared with the general population in terms of occurrence of suicide attempts, and (2) Internal comparison was made within the study population to find out the differences in risk of suicide attempts before, during, and after treatment with Isotretinoin for severe acne. It is essential to monitor isotretinoin users closely for psychiatric effects for at least one year because it has been proven from this study that up to six months after the end of treatment with Isotretinoin, there was an increased risk of a suicide attempt.

The above-discussed studies have proven that some link does seem to exist between the initiation of isotretinoin therapy and the development of psychiatric effects. The effect can be any psychiatric symptom ranging from mild psychosis to suicide attempt. However, the importance of an already existing trigger factor in patients who are started on Isotretinoin is still controversial and needs to be studied further in detail.

Lack of Strong Evidence for an Association Between Isotretinoin Usage and Psychiatric Side Effects

A cohort and a nested case-time-control study were conducted by Droitcourt et al. in France in which 443,814 subjects aged 10-50 years exposed to Isotretinoin were studied using Nationwide French Health Insurance Data from January 1, 2009 to July 31, 2016 [18]. A patient was considered exposed to Isotretinoin from the date of initiation of the drug to the 30th day after the last issue in the same course. The risk of suicide attempts was assessed by conducting two analyses - (1) Observed number of suicide attempts in the isotretinoin population was compared to the expected number in the general population, and (2) Case time control design to spot any isotretinoin initiation-related triggering effect on a suicide attempt. Standardized incidence ratios (SIRs) calculated before, during, and after a course of Isotretinoin were used to analyze the risk of suicide attempts. Standardization was performed on age, gender, month, and calendar year. By using case-time-control analysis, the number of isotretinoin initiations was compared in risk and control periods of two months. The study found that the occurrence of suicide attempts in isotretinoin users was markedly lower than that of the French general population. It also concluded that there was no evidence for a triggering effect of isotretinoin initiation on a suicide attempt.

Erdoğan et al. conducted a prospective, non-randomized, open-label study of three months duration in 2019 with 102 patients [13]. All the patients were adolescents. Out of the 102 patients, 60 were using Isotretinoin, and 42 were using antibiotics. Both the groups were of the same age, gender-matched, same educational level, and had no psychiatric history in family members. The study aimed to assess the quality of life, depression, anxiety, suicide, social anxiety, and obsessive-compulsive symptoms in systemic isotretinoin users. To attain the objective, multiple scales were used in the study, namely Acne Quality of Life Scale (AQLS), Hospital Anxiety and Depression Scale (HADS), Suicide Probability Scale (SPS), Liebowitz Social Anxiety Scale (LSAS), and Maudsley Obsessive-Compulsive Question List (MOCQL). The assessment was done at baseline and after three months. Ultimately, it was found that in acne patients, neither Isotretinoin nor antibiotics affected the levels of depression, anxiety, and suicide. In fact, they were shown to improve the quality of life in patients.

A 12-week longitudinal, open-labeled study was conducted in Venezuela from February 2013 to June 2013 by Suarez et al. on 60 acne patients [15]. They had no previous positive psychiatric history. The subjects were assigned to either the Isotretinoin group (n=36) or to the other treatment group, which consisted of antibiotic treatment (n = 24). Those who had previous exposure/intolerance to Isotretinoin, those below 18 years, and those who refused to participate in the study were excluded. The assessment was done at baseline, six weeks, and 12 weeks of treatment with Zung depression or anxiety scales and two locally developed scales for depression (GeDepr) and anxiety (Ansilet). The "Ge-Depr" is a two-factor scale consisting of 16 depression-related items. The "Ansilet" is a one-factor scale consisting of 15 anxiety-related items. Chi-square test and covariance analysis were used to analyze the data. At the end of the study, it was found that the frequency of depression and anxiety was similar in both groups. Thus, this study confirms the safety of Isotretinoin with respect to psychological effects.

A systematic review and meta-analysis were done by Huang et al. in Thailand to assess the link between Isotretinoin treatment for acne and the risk of depression [20]. Thirty-one studies from PubMed, MEDLINE, Embase, and Cochrane Library databases were taken into account, which met their inclusion criteria. Only the studies that provided the prevalence of depression or depression scores were included in this study. The limitations of the study were - (1) No randomized controlled trials (RCTs) were included, and (2) There was high inter-study variability. The study denied any link between Isotretinoin use and depression. It concluded that Isotretinoin use declined the incidence of depression.

A cohort prospective, questionnaire-based study was conducted in Saudi Arabia by Algamdi et al. from November 2019 to March 2020 on 29 patients [19]. The subjects included in this study were moderate to severe acne patients aged 18-30 years. The subjects excluded were those with a personal history of psychiatric illness, a family history of psychiatric illness, a history of using antipsychotic or antidepressant drugs, a history of receiving a previous course of Isotretinoin, or a history of using recreational drugs. The purpose of the study was to assess the risk of depression in isotretinoin users vs. doxycycline users. The evaluation was done once before starting treatment and later eight weeks after using "patient health-questionnaire 9". Out of the 29 patients, 18 patients, including nine males and nine females, completed the study. Out of the eight, twelve patients received isotretinoin 0.5 mg/kg (study group) and six patients received doxycycline 100 mg (control group). After eight weeks of starting treatment, there was statistically no significant difference in the mean depression score between the two groups. Therefore, the study concluded that no direct relationship exists between the use of Isotretinoin and the development of depression.

The five studies discussed earlier deny the existence of any association between Isotretinoin and psychiatric symptoms.

Improvement in Psychiatric Effects with Isotretinoin Usage

A prospective observational study was done by Nikam et al. in Karad, Maharashtra, India from June 2017 to July 2018 with 300 patients who were more than 12 years old and were taking Isotretinoin [14]. Immunocompromised patients were excluded from the study. The psychiatric effects were evaluated by using the Hamilton anxiety rating scale (HAM-A) and the Montgomery Asberg Depression Rating Scale (MADRS) at baseline up till the fourth visit. Follow-up was done for each patient for a duration of 12 weeks - baseline, first visit after two weeks, second visit after four weeks, third visit after eight weeks, and fourth visit after 12 weeks. During each visit, the changes in the severity of acne were also measured by a visual analog scale (VAS). There was a significant increase in MADRS score at baseline to final visit (P < 0.05) with mild and moderate depression in four (1.3%) and two patients (0.6%), respectively. On the contrary, a significant decrease in HAM-A score was observed over the visits (P < 0.05). The study thus concluded that Isotretinoin caused an improvement in anxiety, whereas there was a negligible effect on depression.

Lastly, the systematic review and meta-analysis done by Li et al. in China conclude that Isotretinoin in acne patients results in improvement of depression symptoms [21]. The discussion included 20 studies from various databases like PubMed, Embase, and Cochrane Library. The language was restricted to English. Quality assessment was done using the nine-star Newcastle-Ottawa scale. The major drawback of this study was that no randomized controlled trial was available. The study suggested that although Isotretinoin might theoretically cause depressive disorders, the depression risk could be compensated by the favorable effects of Isotretinoin on patients with acne. For instance, acne patients tend to get emotionally disturbed, stressed out, and depressed about their physical appearances, which in turn might lead to a series of psychological disorders. Therefore, the use of Isotretinoin may improve the depression caused by the acne itself.

Surprisingly, these two studies went ahead to conclude that Isotretinoin improves psychiatric effects in acne patients.

Therefore, we have one section of the researchers stating an association between Isotretinoin and psychiatric side effects in acne patients, while the other section denies such a link. 

Limitations

One of the weaknesses of this paper is that many of the studies included lack adequate sample size. It would be better and more accurate to assess a population with a large sample size for a longer period. The other limitation is the absence of randomized controlled trials pertaining to this research question. We need more research trials in the future to see to what extent the benefits of isotretinoin use outweigh the psychiatric side effects.

## Conclusions

We aimed to study the association between isotretinoin use and psychiatric side effects in acne patients and whether pre-existing risk factors altered the occurrence of such outcomes. Isotretinoin is a fat-soluble drug that can cross the blood-brain barrier and, therefore, cause neurological changes. Our research found from a few studies that Isotretinoin does have some connection with psychiatric side effects (primarily depression and suicide attempts). In patients with a pre-existing psychiatric history, the chances of suicide attempts are found to be more. However, since it is unclear why only some cases and not all are affected by this drug, we conclude that it is alright to prescribe this drug for acne after a thorough evaluation of risk factors. This study's benefit is that health care workers and patients should keep in mind that the possibility of occurrence of neuropsychological side effects due to Isotretinoin cannot be ruled out. One of the future recommendations is that further research on this topic should be done to figure out why one section of the users gets these effects whereas the other section does not. A clear-cut reason for this difference should be determined by investigating this topic more thoroughly using larger sample sizes for more extended periods.
